# FhlA-mediated regulation of proton transport and energy transduction in *Escherichia coli* at acidic pH

**DOI:** 10.1371/journal.pone.0351220

**Published:** 2026-06-25

**Authors:** Heghine Gevorgyan, Anait Vassilian, Anna Poladyan, Karen Trchounian

**Affiliations:** 1 Department of Biochemistry, Microbiology and Biotechnology, Faculty of Biology, Yerevan State University, Yerevan, Armenia; 2 Research Institute of Biology, Faculty of Biology, Yerevan State University, Yerevan, Armenia; 3 Microbial Biotechnologies and Biofuel Innovation Center, Yerevan State University, Yerevan, Armenia; Federal University Dutse, NIGERIA

## Abstract

In *Escherichia coli,* the FhlA acts as a transcriptional activator for *fdhF*, *hyc* and *hyp* operons, whose gene products constitute the formate hydrogen lyase (FHL) complexes. These complexes contribute to bioenergetic regulation by consuming intracellular protons during the conversion of formate to hydrogen gas (H_2_). The current study elucidates the role of the FHL complex (using *fhlA* mutant) in the proton transport and energy transduction during the fermentation of glucose, glycerol and formate at pH 5.5. It was shown that F_O_F_1_-ATPase activity and proton flux rate were decreased in *fhlA* at 20 h and 72 h grown cells, compared to WT indicating the functional interplay between FHL and F_O_F_1_ and highlighting its importance in proton transfer at acidic conditions. Increased number of membrane –SH groups and decreased proton conductance (C_M_H^+^) value in both WT and *fhlA* mutant were detected at 72 h, compared to 20 h, suggesting efficient energy transduction at acidic pH when H_2_ was not generated. The value of membrane potential (ΔΨ) remained unchanged in FhlA-lacking cells and was independent of growth time. Thus, bacteria regulate proton motive force (Δp) managing bioenergetic association between proton ATPase activity, FHL function and transmembrane proton gradient (ΔpH) regulation. Overall, these findings demonstrate that the FHL complex plays an essential role in coordinating proton flux, regulation of proton motive force and energy transduction in *E. coli* under acidic fermentative conditions through functional interplay with the F_O_F_1_-ATPase.

## Introduction

*Escherichia coli* is a facultative anaerobic bacterium that survives in diverse environmental conditions [1]. Among these, extracellular pH is a critical factor influencing physiological parameters and metabolic regulation including energy metabolism. Under acidic conditions, *E. coli* requires precise regulation (fine tuning) of bioenergetic processes to maintain cellular homeostasis. Neutrophilic bacteria mobilize specific protective mechanisms by which they respond to acidic conditions [2,3]. At low pH, maintaining intracellular pH (pH_in_), energy production, and membrane integrity becomes vital, as proton gradients might be altered [2,4,5]. The formate hydrogen lyase (FHL) complex is a membrane-bound system that couples formate oxidation to molecular hydrogen (H₂) production in *E. coli* during anaerobic fermentative conditions involved in acid resistance mechanisms [3,6,7].

In *E. coli*, formate oxidation to CO_2_ and H^+^ occurs via cytoplasmic formate dehydrogenase (Fdh-H) [8–10]. Subsequently, hydrogenase-3 (Hyd-3) in FHL-1 and hydrogenase-4 (Hyd-4) in FHL-2 catalyze H^+^ to H_2_ reaction [10,11]. By consuming two protons per H_2_ molecule formed, the FHL complex contributes to the regulation of pH_in_ and proton motive force (Δp) [12]. The expression and activity of the FHL complex is regulated by various environmental factors [6,13]. Two regulatory proteins, FhlA and HycA, play critical role in modulating the expression and function of the FHL system. Transcriptional activator FhlA integrates environmental signals such as formate availability and pH. FhlA protein is a transcriptional activator for *fdhF*, *hyc* and *hyp* operons and is found in the cytoplasm [14,15].

It has been demonstrated that the FhlA regulatory protein controls the transcription of enzymes contributing to metabolic fluxes formation at acidic (pH 5.5 and pH 6.5) and alkaline (pH 7.5) pH values [6,12,16]. The role of the FhlA transcriptional activator in modulating proton flux and proton conductance has been particularly highlighted at pH 7.5. Moreover, FHL complexes transfer protons generated from formate to F_O_F_1_ for energy conservation at alkaline pH. FhlA-lacking cells decrease proton conductance and the total rate of H^+^ flux for efficient energy transduction and maintaining Δp. [17]. However, the bioenergetic consequences of the FHL complex’s activity and its regulation remain poorly understood, particularly under acidic conditions where proton gradients and membrane energetics are substantially altered, compared to alkaline conditions.

The involvement of the proton F_O_F_1_-ATPase is also considered to contribute to tolerance of acidic conditions [18–20]. During anaerobic fermentative conditions, F_O_F_1_ functions in the ATP hydrolysis mode pumping protons out of the cell [19]. Moreover, Δp is generated and maintained as a function of proton ATPase. From another point of view, H_2_ cycling via Hyds is suggested as an alternative pathway to maintain Δp [19,21]. When pH_in_ decreased, proton ATPase is used for pumping protons out using ATP energy. In addition, generated organic acids might dissipate proton motive force (Δp). Organic acids dissipate Δp mainly by acting as proton carriers, collapsing the ΔpH component. Some of them indirectly affect membrane potential (ΔΨ). [19,22]. The impact of weak organic acids on the physiological, biochemical and bioenergetic parameters of bacteria is not only due to the variation of pH transmembrane gradient (ΔpH), but also the specific impact they have on cytoplasmic or membrane-bound enzymes [23,24]. ΔpH is a component of Δp and its alteration might lead to compensatory changes in the value of ΔΨ to maintain Δp [25]. The generation of Δp depends on environmental conditions like pH and oxidation reduction potential, and regulation of Δp and proton permeability in the membrane is essential for energy transduction and cellular homeostasis [26,27]. Redox potential influences Δp indirectly by controlling the redox reactions (fermentation pathways, ATP yield) that generate or consume Δp. From this perspective, proton conductance and permeability directly influence Δp, which, in turn, provide a driving force to H^+^ movement.

The main objective of the current work is to elucidate the mechanistic role of the FhlA regulator in coordinating the functional interplay between the FHL complex and the F_O_F_1_-ATPase system, with particular emphasis on proton translocation, energy conservation, and redox balance under acidic fermentative conditions (pH 5.5). Two time points were chosen for experiments to represent distinct physiological states of bacterial growth under anaerobic fermentative conditions: 20 h corresponds to the active fermentation phase when bacteria consumed glucose, whereas 72 h represents a late-stage or prolonged fermentation condition, when glycerol was remained as a carbon substrate, redox balance was altered, and hydrogen production was absent. These findings advance our understanding of bacterial physiology and adapting mechanism at acidic environments clarifying the role of the FHL complex in maintaining pH and energy homeostasis.

## Results

To gain a comprehensive understanding of the bioenergetics of *E. coli* at pH 5.5 and the role of FhlA protein in the formation in bioenergetic properties, several key parameters were analyzed: proton ATPase activity, the availability of accessible sulfhydryl (-SH) groups, ∆pH (the transmembrane pH gradient), membrane potential (ΔΨ), and the proton motive force (Δp). Together, these parameters provide insights into energy conservation and proton fluxes under acidic pH. All measurements were done at 20 h and 72 h grown cells on the mixture of glucose, glycerol and formate and the utilizing substrate was changed: at 20 h the utilizing substrate was glucose and after 20 hours, when there was no glucose available, *E. coli* start consuming glycerol (72 h) [6,12].

### Proton F_O_F_1_-ATPase activity and H^+^ flux rate

**Fig 1:**
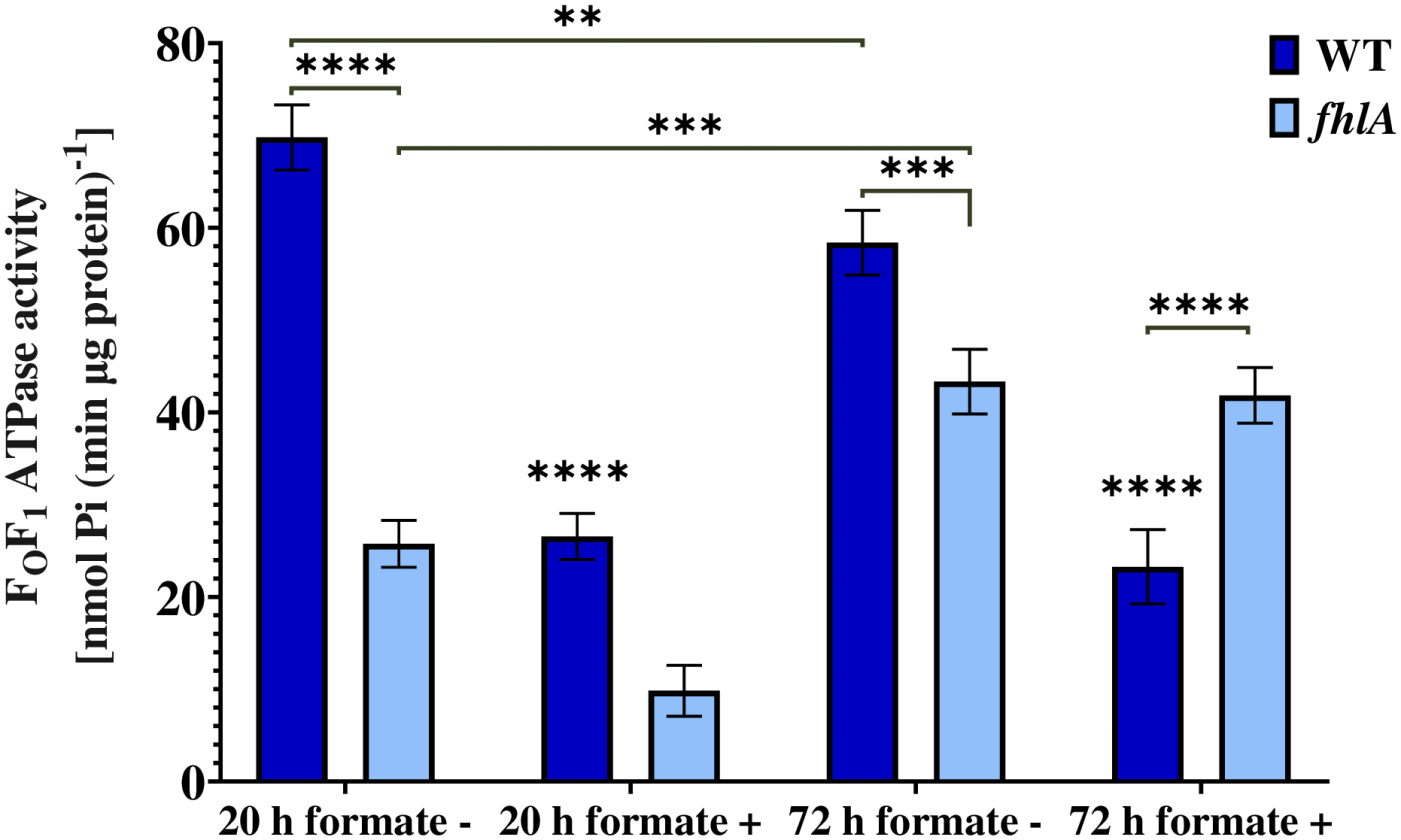
F_O_F_1_-ATPase activity of membrane vesicles of *E. coli* WT and *fhlA* at 20 h and 72 h. F_O_F_1_-ATPase activity was calculated as a difference between the values of overall and DCCD assays. The DCCD (0.1 mM) was added into the assay medium. Formate (0․68 g L^-1^) added in the assays indicated as formate + . Bacteria were grown during fermentation of mixed carbon sources (glucose (2 g L^-1^), glycerol (10 mL L^-1^) and formate (0.68 g L^-1^)). Significance (p < 0.05) was determined by Tukey’s multiple comparison test. Data are represented as mean ± SD. ns: not significant, ****p < 0.0001, ***p < 0.001, **p < 0.01, n = 3.

Proton ATPase has important contribution in the *E. coli* survival mechanism at pH 5.5 [9,20,28]. To understand the role of FhlA-regulated FHL complex in the proton ATPase activity F_O_F_1_-ATPase activity was investigated at 20 and 72 hours during the fermentation of the carbon mixture (Fig 1). F_O_F_1_-ATPase activity was shown to be ~ 70 nmolP_i_ (min μg protein)^−1^. The enzyme activity was lower by ~ 65% in the mutant strain at 20 h, compared to WT. F_O_F_1_-ATPase activity was decreased by ~ 20% in WT at 72 h, compared to the value at 20 h. On the contrary, in the *fhlA* mutant strain, proton ATPase activity was lower ~ 25%, compared to WT and was higher by ~ 40% compared to the activity at 20 h.

**Fig 2:**
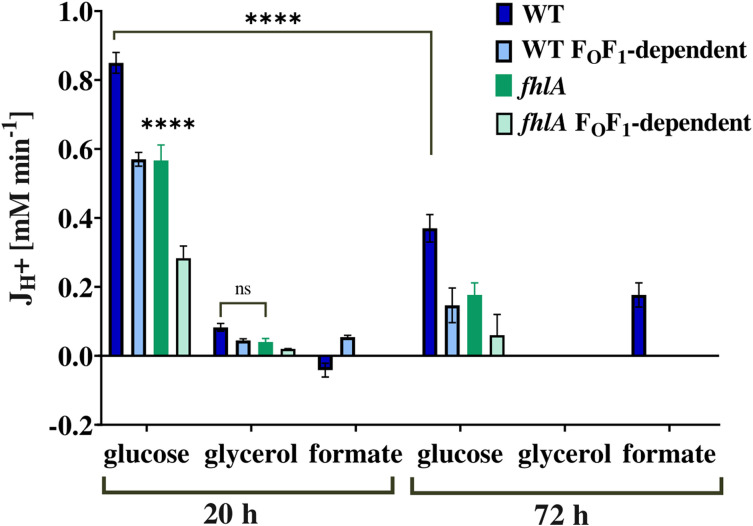
H^+^ flux rate (J_H+_) in whole cells of *E. coli* WT and *fhlA* at 20 h and 72 h. In assays, substrates were used in the same concentration as in the growth medium. F_O_F_1_-dependent J_H+_ was calculated as a difference between the values of overall and DCCD assays. The DCCD (0.1 mM) was added into the assay medium. Bacteria were grown during fermentation of mixed carbon sources (glucose (2 g L^-1^), glycerol (10 mL L^-1^) and formate (0.68 g L^-1^)). Significance (p < 0.05) was determined by Tukey’s multiple comparison test. Data are represented as mean ± SD. ns: not significant, ****p < 0.0001, n = 3.

H^+^ flux rate was ~ 0.85 mM min^-1^ in WT at 20 h in glucose supplemented assays, which was decreased in *fhlA* by ~ 35% (Fig 2). When glycerol was added to the experimental medium H^+^ flux rate in WT was ~ 0.08 mMmin^-1^ at 20 h. During formate addition in the assay, H^+^ outflux was not detected. At 72 h, H^+^ flux rate was decreased in the glucose-supplemented assay in WT and *fhlA* by ~ 55% and ~ 70%, respectively, compared to 20 h. H^+^ flux was not detected when glycerol was added to the experimental medium. Meanwhile, during formate addition, the H^+^ flux rate rate was 0.18 mM min^-1^.

### Redox potential and Accessible -SH groups

Redox potential was detected during anaerobic fermentative growth [16]. It was shown that Redox potentialwas decreased to negative values (−500 mV) in WT at 20 h showing the H_2_ generation in WT [29,30]. Meanwhile, in the *fhlA*, where H_2_ production was not detected, Redox potential values dropped to −175 mV at 20 h.

**Fig 3:**
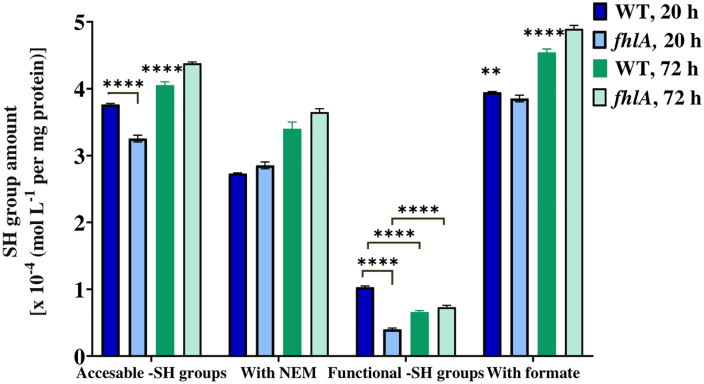
SH group amount in membrane vesicles of *E. coli* WT and *fhlA* at 20 h and 72 h. The NEM (0.1 mM) was added into the assay medium. Formate (0.68 g L^-1^) was added to the assays. Bacteria were grown during fermentation of mixed carbon sources (glucose (2 g L^-1^), glycerol (10 mL L^-1^) and formate (0.68 g L^-1^)). Significance (p < 0.05) was determined by Tukey’s multiple comparison test. Data are represented as mean ± SD. ****p < 0.0001, **p < 0.01, n = 3.

The amount of accessible –SH groups was ~ 3.8 x 10^−4^ mol L^-1^ per mg protein in WT at 20 h, which was decreased in the *fhlA* reaching ~3.2 x 10^−4^ mol L^-1^ per mg protein (Fig 3). In the presence of N-ethylmaleimide (NEM), which is known as a thiol group-specific modifier, the amount of -SH groups was decreased [31]. The amount of functional -SH groups (difference between overall and NEM-influenced -SH amount) was also reduced in the *fhlA* mutant ~2.5 fold at 20 h. Moreover, the amount of overall accessible –SH groups was higher at 72 h in both strains. However, the amount of functional -SH groups decreased in wt ~ 1.6 fold, and increased in *fhlA* ~ 1.9 fold, compared to 20 h.

### Δp generation and proton conductance

pH_in_ and pH_ex_ were determined at 20 and 72 hours of the growth (Table 1). pH_in_ was ~ 6.56 at 20 h in WT, which was not significantly changed in the *fhlA*. pH_ex_ was ~ 5.10 in the WT and mutant cells at 20 h. Strict variation of pH_in_ and pH_ex_ was detected at 72 h. The alkalinization of pH_in_ was detected by ~ 0.2 units in WT and *fhlA*. pH_ex_ was decreased by 0.4 units at 72 h in the *fhlA* mutant, compared to WT. As a result of the variation of pH_in_ and pH_ex_ the values of ΔpH were changed (Table 1).

**Table 1:** The values of pH_in_, pH_ex_, ΔpH, Δψ, Δp and C_M_H^+^ in *E. coli* WT and *fhlA* at 20 and 72 hours of the growth in the presence of a mixture of glucose, glycerol and formate.

	20 h		72 h	
	WT	*fhlA*	WT	*fhlA*
**pH**_**in**_	6.56 ± 0.020	6.60 ± 0.017	6.75 ± 0.021	6.80 ± 0.03
**pH**_**ex**_	5.10 ± 0.012	5.03 ± 0.01	5.32 ± 0.013	4.92 ± 0.013
**61.5ΔpH** **(mV)**	−89 ± 0.71	−97 ± 0.62	−88 ± 0.98	−116 ± 2.5
**ΔΨ** **(mV)**	−110 ± 3.2	−110 ± 3.0	−113 ± 2.3	−110 ± 3.0
**Δp** **(mV)**	−199 ± 3.4	−207 ± 3.4	−201 ± 3.3	−225 ± 1.2
**C**_**M**_**H**^**+**^ **(nM min**^**-1**^ **mV**^**-1**^ **10**^**9**^ **cells)**	4.25 ± 0.11	2.75 ± 0.06	1.84 ± 0.06	0.79 ± 0.01

*At 37°C, 2.3RT/F equals ~61.5.

However, alteration in the value of ΔΨ was not detected during the growth in both WT and FhlA-lacking cells. As a result, Δp was changed negligibly in the *fhlA* mutant strain at 20 h (from −199 mV to −207 mV) and was increased by ~57 mV at 72 h (from −201 mV to −225), compared to WT (Table 1).

Proton conductance was 4.25 mM min^−1^ mV^-1^ per 10^9^ cells in WT at 20 h (Table 1), which decreased by ~ 35% in *fhlA*. Meanwhile, at 72 h proton conductance decrease reached to 1.84 and 0.79 nM min^−1^ mV^-1^ per 10^9^ cells in WT and *fhlA*, accordingly.

## Discussion

### Proton transport and energy transduction in the cells grown at 20 h

F_O_F_1_-ATPase activity and other Δp generating parameters are changed in the *E. coli* cells lacking regulatory FhlA protein. F_O_F_1_-ATPase activity was decreased in the mutant cells. It is suggested that H^+^ generated via FHL pathway might transport to F_O_F_1_ at 20 h (Fig 1). This is consistent with previous observations indicating that FHL component FDH-H interplays with F_O_F_1_ during the fermentation of glucose, glycerol and formate [9,28]. Moreover, our previous studies suggested the interaction between F_O_F_1_ and Hyds at 20 h using *hypF* mutant during fermentation of mixed carbon sources [28]. Proton transfer from the FHL complex to the F_O_F_1_-ATPase is proposed to occur via Fdh-H–Hyd–F_O_F_1_ and/or Fdh-H– F_O_F_1_ and/or Fdh-H–Hyd–H_2_ pathways, contributing to energy conservation. [9,20]. This is complemented by the decrease of H^+^ flux rate in the *fhlA* strain (Fig 2). The proposed disulfide/dithiol (–S-S- to –SH HS-) interchange between F_O_F_1_ and FdhH and/or other components of the FHL complex, as overall and functional –SH groups were decreased in both *fdhF* and *fhlA* strains [9] (Fig 3). Furthermore, overall and F_O_F_1_-dependent H^+^ flux rate was decreased in the *fhlA* (Fig 2). Thus, when the FHL complex was not functional, “additional” H^+^ was not transported to the F_O_F_1_, resulting in decreased H^+^ flux rate which was detected in parallel to the reduction of F_O_F_1_-ATPase ATPase activity. Alternatively, it is possible that H^+^ can efflux via FHL components like the Hyd-4 HyfF subunit [32]. Alternatively, formate may not be efficiently neutralized in the mutant, leading to its accumulation in the cytoplasm or its efflux into the external environment [16]. It is important to mention that external formate reduced the F_O_F_1_-ATPase activity, suggesting that excess quantity of formate: external or internal (generated during fermentation), decreased the F_O_F_1_-ATPase activity and proton flux rate. This way, bacteria maintained intracellular pH more stable. Besides, the addition of formate to the reaction mixture reduced F_O_F_1_-ATPase activity in WT and *fhlA* similarly (by ~60%), proton flux was absent and the amount of accessible –SH groups are not changed in WT suggesting that external formate might have a direct influence on the proton F_O_F_1_-ATPase activity.

pH_in_ was not significantly changed in the mutant strain, compared to WT. When F_O_F_1_ is less active in the *fhlA*, it is suggested that other systems, such as acid resistance systems might work efficiently to regulate pH_in_ [3,33]. It is proposed that that F_O_F_1_ is not the main proton effluxing and pH_in_ regulating system at pH 5.5 and the ATP-dependent system plays an important role in the survival of *E. coli* under acidic conditions in addition to amino acid decarboxylation based acid resistance systems [5].

Redox potentialwas more reduced in WT conditioned by the generation of H_2_ [29]. In the *fhlA* cells enzymes catalyzing the H^+^ to H_2_ reaction are absent. Due to that H_2_ was not generated resulting in more oxidized Redox potential [16]. Thus, pH_ex_ was reduced as a result of H_2_ absence and formate accumulation in the extracellular environment (Table 1) [16]. Substantial variation of ΔΨ and Δp was not detected in the *fhlA*, compared to WT. Assuming that regulation of the Δp occurred via F_O_F_1_-ATPase and H^+^ flux rate variation, in which the role of FHL components is evident at acidic pH 5.5. Moreover, proton conductance was decreased in the *fhlA* suggesting efficient energy transduction in the mutant cells, when one of the Δp generating system (Hyds) is not functional.

In our conditions, proton ATPase and FHL complex have an interplay suggesting energy conservation under fermentative conditions [6,19]. H^+^ to H_2_ reaction might be occurred in the membrane via the dithiol-disulphide interchange: 2SH - > –S-S- + H_2_. The reduced state of dithiol is suggested to be energy or proton “storehouse”, deprotonation of which leads to energy release [34,35]. Thus, local transduction of energy within the F_O_F_1_ and FHL components was suggested. And, in the *fhlA* mutant, the amount of –SH groups was decreased indicating that there is no need for the conservation of energy because these cells are not able to generate H_2_.

Moreover, as proton conductance was decreased in the *fhlA*, suggesting that at least one H^+^ for H_2_ generation might be provided from F_O_F_1_ via dithiol/disulfide interchange using –SH liberalization energy [35].

### Regulation at 72 hour

The relatively small difference between F_O_F_1_-ATPase activity at 20 and 72 hours in WT and the decrement of total H^+^ flux rate at 72 h (Figs 1 and 2) can be a result of the slow utilization rate of glycerol by cells at 72 h [16,20]. Moreover, it was shown that the rate of H^+^ efflux by *E. coli* during glycerol fermentation was much lower than that during glucose fermentation [21]. It is likely aimed at regulating ΔpH at 20 and 72 hours indicating the function of F_O_F_1_-ATPase in Δp [19]. The opposite phenomenon in the *fhlA* mutant was due to formate accumulation and H_2_ absence (pH_ex_ was lower, F_O_F_1_-ATPase activity was higher), which alkalized the internal medium to regulate ΔpH. Moreover, F_O_F_1_-ATPase activity was decreased in WT and was increased in FhlA-lacking cells at 72 h when external formate was added to the reaction mixture (Fig 1). Previously was shown that WT cells displayed FDH-H activity at 72 h [36]. Alternatively, as H_2_ generation was absent at 72 h, H^+^ efflux might occur via the FHL components, besides F_O_F_1_. Moreover, in FhlA-lacking cells, when FHL complexes were not synthesized, formate might directly influence the proton ATPase activity for the H^+^ flux as a coupling system with lactate efflux and formate absorbance at 72 h [16]. These findings reveal the translocation of protons via F_O_F_1_, and the functional interplay of proton ATPase activity, FHL function and ΔpH regulation at acidic pH.

*E. coli* cells increased pH_in_ when pH_ex_ was decreased (Table 1). This way, bacterial cells provide a high value of ΔpH to survive at acidic pH. ΔpH was unchanged in *fhlA* at 20 h due to the regulation of acid ratio in bacterial growth media. However, at 72 h, ΔpH was increased indicating the role of FHL complexes in the regulation of ΔpH (discussed above). Variation in ΔΨ was not detected in WT depending on the growth time and utilizing substrate. Moreover, FHL complexes did not have a contribution to the ΔΨ generation. Thus, changes in the value of Δp at 72 h were due to the alterations in ΔpH. Similar results were shown at pH 7.5 [6].

Proton conductance was decreased in WT at 72 h suggesting efficient energy transduction when bacteria slowly utilized glycerol, the activity of proton ATPase and H^+^ flux rate was decreased, compared to 20 h. Moreover, the Redox potentialvalue was more oxidized (by ~ −180 mV) at 72 h conditioned by the H_2_ absence during glycerol fermentation [30]. Previously was shown that when Redox potential was higher, proton conductance was decreased and the dithiol/disulfide balance was changed [37]. Functional –SH groups in the membrane were more oxidized (–S-S-) at 72 h, compared to 20 h, and bioenergetic association between FHL components in the F_O_F_1_-ATPase might decrease. In addition, H^+^ flux rate was decreased at 72 h suggesting decreased H^+^ permeability during glycerol utilization in the mixture [37].

The amount of functional –SH groups were decreased in WT and increased in the *fhlA*, compared to 20 h (Fig 3). Moreover, there is no significant changes between WT and *fhlA* at 72 h. This phenomenon is a result of the absence of H_2_ at 72 h, when glycerol is consuming. From another point of view, formate addition in the reaction mixture increased the value of accessible –SH groups [9,38]. Via this way, when formate was consumed at 72 h in *fhlA*, it might increase the –S-S- to –SH formation in the membrane. This phenomenon was not shown at 20 h as in *fhlA* mutant formate was effluxed out, probably to regulate ΔpH. Moreover, proton conductance was decreased in *fhlA* indicating efficient energy transduction. Thus, suggested that bacteria perform –S-S- to –SH reaction to save protons or energy (from formate) in the membrane when H_2_ generation was absent and H^+^ flux rate was lower.

## Conclusions

**Fig 4:**
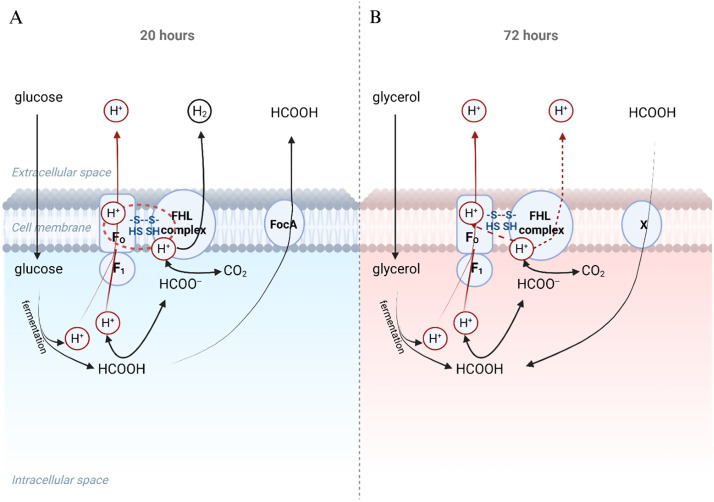
Proposed mechanisms underlying the functional interplay between FHL complex and F_O_F_1_-ATPase in *E. coli* during fermentative growth at pH 5.5. **(A)** At 20 h, corresponding to active glucose fermentation, the FHL complex is functional and catalyzes formate oxidation and H₂ production. Protons (H⁺) generated from formate dissociation are proposed to be transferred to the F_O_F_1_-ATPase via direct or indirect coupling pathways, possibly involving dithiol/disulfide exchange, forming a cyclic process that supports proton flux and energy conservation. **(B)** At 72 h, corresponding to prolonged glycerol fermentation, FHL in not active and H₂ is not produced. Under these conditions, protons derived from formate are proposed to be redirected primarily toward the F_O_F_1_-ATPase and/or released into the extracellular medium, reflecting altered proton transport pathways and reduced coupling between FHL and ATPase activity. The figure is adapted to [11,16,20,32,35,51,52]. The figure was created with BioRender.com.

In the current work we tried to explain the core concept of proton transport, formation of bioenergetic parameters and energy transduction mechanism in *E. coli fhlA* lacking cells during mixed carbon fermentation at acidic pH 5.5 depending on the utilizing substrate (Fig 4). The FhlA protein is a key regulator that not only controls FHL synthesis but also dictates the functional efficiency of the proton ATPase through a synergistic interaction. At 20 h, when cells utilize glucose, proton transfer from the FHL complex to F_O_F_1_-ATPase is proposed to occur via the Fdh-H – Hyd – F_O_F_1_ and/or Fdh-H – F_O_F_1_ and/or Fdh-H – Hyd – H_2_ [20]. Similar interaction was determined also at 72 h, however, when H_2_ was not generated, H^+^ is suggested to be effluxed via components of FHL complex [20]. Moreover, it was shown a membrane-based “energy storehouse” where dithiol-disulfide transitions (2 SH to -S-S-) facilitate local energy transduction between the FHL complex and the proton ATPase, besides, the important role of formate in the functional -SH groups was hypothesized. Formate acts as a regulator of F_O_F_1_-ATPase activity. Its accumulation in the absence of a functional FHL complex might serve as a signal to reduce proton outflux to maintain pH_in_ stability. At pH 5.5 during anaerobic fermentative conditions, *E. coli* prioritizes the variation of ΔpH over ΔΨ. This is achieved by coordinating FHL activity, F_O_F_1_-dependent proton flux, and membrane conductance to optimize energy conservation. The shift from 20 h to 72 h demonstrates the bacteria’s ability to reconfigure its redox state and membrane thiol accessibility to adapt to the depletion of primary substrates and the accumulation of metabolic end-products.

## Materials and methods

### Bacterial strains and growth conditions

*E. coli* BW 25113 WT *(rrnB* Δ*lacZ4787 HsdR514* Δ*(araBAD)567* Δ*(rhaBAD)568 rph-1* [39] and JW2701 (*ΔfhlA*) mutant [40] defective in FHL activator strains from Keio collection were used.

Bacteria were grown in the highly buffered peptone medium comprising 20 g L^-1^ peptone, 1.08 g L^-1^ K_2_HPO_4_, 15 g L^-1^ KH_2_PO_4_, 5 g L^-1^ NaCl (pH 5.5) [9,28]. Glucose (2 g L^-1^), glycerol (10 mL L^-1^), and sodium formate (0.68 g L^-1^) were added to the medium as carbon sources. Overnight anaerobically grown cultures were transferred into the growth medium and were incubated anaerobically at 37^o^C for 20 h and 72 h. The initial pH of the medium was determined using a pH meter with a selective pH electrode (HI1131B, Hanna Instruments, USA) and adjusted to pH 5.5 using 0.1 M HCl before inoculation, then was not regulated. The medium was prepared in glass vessels, with oxygen removed by bubbling during autoclaving, after which the vessels were sealed with plastic press caps as previously described [41]. Samples were collected via the sterile syringe.

### Membrane vesicles and proton ATPase activity

*E. coli* cells were harvested at 20 and 72 hours of growth. Membrane vesicles (MV) were obtained from bacteria by inducing the osmotic lysis of spheroplasts through the treatment with lysozyme and ethylenediaminetetraacetic acid (EDTA) [28,42,43].

The ATPase activity was determined by measuring the amount of inorganic phosphate (P_i_) generated in the reaction of membrane vesicles with 5 mM ATP. The reactions were conducted in 50 mM Tris-HCl buffer at 37°C, with the pH adjusted to match the respective growing environment (pH 5.5). ATPase activity was quantified in nmol P_i_ (min μg protein)^−1^. P_i_ was determined spectrophotometrically (UV–VIS spectrophotometer, Cary 60, Agilent Technologies, USA) as described [9,28].

To determine F_O_F_1_-ATPase activity *N,N’-* dicyclohexylcarbodiimide (DCCD) with 0.1 mM of final concentration was used. The F_O_F_1_-ATPase activity was calculated as a difference between activities in the absence and in the presence of the inhibitor. To study the effect of formate in the assays 10 mM sodium formate was added when indicated. MV were incubated with formate for 10 min. All assays were done at 37^0^C. Protein levels were measured by the method of Lowry et al. (1951) using bovine serum albumin (BSA), as a standard [44].

### Redox potential and Accessible SH groups

Redox potential (E_h_, mV) was measured during the bacterial growth by Pt redox sensitive electrode (HI3131B electrode, HANNA Instruments, USA). Redox potentialvalue for H^+^/H_2_ conversion during anaerobic growth is −414 mV indicating H_2_ production [29]. This methodology shares similarities with the Clark-type electrode utilized by Noguchi et al. [45]. Moreover, this approach is close to the method employed by Fernandez [46] showing similarity of the H_2_ determination methods (amperometric, chromatographic and electrochemical).

SH-groups were determined by the reaction with Ellmann’s reagent, as described [47] using glutathione as a standard. MVs were treated with the reagent until the latter was fully reacted, and the optical density became constant. To quantify accessible –SH groups N-ethylmaleimide (NEM) was used with 0.1 mM final concentration. The level of SH-groups was expressed in 10^−4^ mol L^-1^ per mg protein.

### Rate of proton flux

The rate of proton flux (J_H+_) in whole cells was determined in the assays using 150 mM Tris–phosphate buffer containing 0.4 mM MgSO4, 1 mM NaCl, 1 mM KCl (pH 5.5) [32,48]. Glucose, glycerol, or formate were added to the buffer at the same concentrations as in the growth medium. After the addition of the bacterial suspension, the proton levels were measured using an ion-meter (HI 5222, Hanna Instruments, USA) equipped with a proton-sensitive electrode (HI1131B electrode, Hanna Instruments, USA) in the external medium. The electrode readings were calibrated by titration of the medium with 0.01 M HCl. J_H+_ was expressed in mM min^−1^ per 10^9^ cells.

### Proton motive force and proton conductance

Extracellular pH (pH_ex_) was measured via a pH-meter with a selective pH-electrode (HI1131B, Hanna Instruments, USA). Intracellular pH (pH_in_) was determined using 9-aminoacridine fluorescent dye (9-AA, with excitation at 339 nm and emission at 460 nm) [49]. 9-AA is distributed across the membrane according to ΔpH, the uptake of which by bacterial cells was determined from the quenching of fluorescence (Cary Eclipse, Agilent Technologies, USA). ΔpH was calculated as the difference between pH_in_ and pH_ex_, as described elsewhere [50].

Membrane potential (ΔΨ) inside negative was measured determining tetraphenylphosphonium cation (TPP^+^) distribution between the bacterial cytoplasm and the external medium at a steady state level, as described elsewhere [6,43]. The assay was done in a thermo-stated vessel of 4 mL with 150 mM Tris–HCl buffer pH 5.5 containing 1 μM TPP^+^. The changes in the TPP^+^ concentration in the external medium were determined by using a TPP^+^-selective electrode. The absorption of TPP^+^ on the bacterial cell surface was determined for boiled (during 3 min) cells [41].

Δp was calculated as a sum of Δψ and ΔpH according to Δμ_H+_/F = ΔΨ − ZΔpH (negative value in mV), where Z is RT/F equal to 61.1 mV at 37°C [50]. Proton conductance of the membrane (C_M_H^+^) was calculated by the following formula: C_M_H^+^= J_H+_/Δp as described [25,41]. C_M_H^+^ was expressed in nM min^−1^ mV^-1^ per 10^9^ cells.

### Others, reagents and data processing

Protein levels were measured by the method of Lowry [44] using bovine serum albumin (BSA), as a standard. MVs were incubated with 0.1 mM DCCD (ethanol solution) for 10 min prior assays; ethanol in the final concentration of 0.5% was used, as a blank; no effect of ethanol in the used concentration on growth and ATPase activity was observed. In assays where formate (10 mM) is added it was described as formate assays. All assays were done at 37^0^C.

Agar, peptone, glycerol, sodium formate, Tris (Carl Roth GmbH, Germany), ATP, BSA, DCCD, lysozyme (Sigma-Aldrich, Germany) and other reagents of analytical grade were used.

### Statistics

Average data obtained from three independent cell cultures are represented and standard deviations of values do not exceed 3% if not given. Results are presented as mean ± SD. A p-value of less than 0.05 was considered significant. Data were visualized using GraphPad Prism 8 software. Significance (p < 0.05) was determined by two-way ANOVA and Tukey’s multiple comparisons test for F_O_F_1_-ATPase activity, H^+^ flux rate, Redox potentialand Amount of –SH groups. Average data obtained from 3 independent assays are represented [41].

## Supporting information

S1 Data Raw data for Fig 1. F_O_F_1_-ATPase activity of membrane vesicles of *E. coli* WT and *fhlA* at 20 h and 72 h. F_O_F_1_-ATPase activity was calculated as a difference between the values of overall and DCCD assays. The DCCD (0.1 mM) was added into the assay medium. Formate (0․68 g L^-1^) added in the assays indicated as formate + .
(XLSX)

S2 Data Raw data for Fig 2. H^+^ flux rate (J_H+_) in whole cells of *E. coli* WT and *fhlA* at 20 h and 72 h. In assays, substrates were used in the same concentration as in the growth medium. F_O_F_1_-dependent J_H+_ was calculated as a difference between the values of overall and DCCD assays. The DCCD (0.1 mM) was added into the assay medium.
(XLSX)

S3 Data Raw data for Fig 3. –SH group amount in membrane vesicles of *E. coli* WT and *fhlA* at 20 h and 72 h. The NEM (0.1 mM) was added into the assay medium. Formate (0.68 g L^-1^) was added to the assays.
(XLSX)

S4 Data Raw data for Table 1. The values of pH_in_, pH_ex_, ΔpH, Δψ, Δp and C_M_H^+^ in *E. coli* WT and *fhlA* at 20 and 72 hours of the growth in the presence of a mixture of glucose, glycerol and formate.
(XLSX)

## Abbreviations

FhlA: transcriptional activator for *fdhF*, *hyc* and *hyp* operons
FDH-H: formate dehydrogenase H
Hyd: Hydrogenase
ΔΨ: membrane potential
Δp: proton motive force
pH_ex_: extracellular pH
pH_in_: intracellular pH
ΔpH, pH_in_, pH_ex_: tansmembrane proton gradient
